# Advancements in Monitoring Water Quality Based on Various Sensing Methods: A Systematic Review

**DOI:** 10.3390/ijerph192114080

**Published:** 2022-10-28

**Authors:** Siti Nadhirah Zainurin, Wan Zakiah Wan Ismail, Siti Nurul Iman Mahamud, Irneza Ismail, Juliza Jamaludin, Khairul Nabilah Zainul Ariffin, Wan Maryam Wan Ahmad Kamil

**Affiliations:** 1 Advanced Devices and System, Faculty of Engineering and Built Environment, Universiti Sains Islam Malaysia, Nilai 71800, Negeri Sembilan, Malaysia; 2 TF AMD Microelectronics Sdn Bhd, Kawasan Perindustrian Bayan Lepas, Bayan Lepas 11900, Pulau Pinang, Malaysia; 3 School of Physics, Universiti Sains Malaysia, Gelugor 11800, Pulau Pinang, Malaysia

**Keywords:** embedded sensors, water quality monitoring system, water pollution and sensing methods

## Abstract

Nowadays, water pollution has become a global issue affecting most countries in the world. Water quality should be monitored to alert authorities on water pollution, so that action can be taken quickly. The objective of the review is to study various conventional and modern methods of monitoring water quality to identify the strengths and weaknesses of the methods. The methods include the Internet of Things (IoT), virtual sensing, cyber-physical system (CPS), and optical techniques. In this review, water quality monitoring systems and process control in several countries, such as New Zealand, China, Serbia, Bangladesh, Malaysia, and India, are discussed. Conventional and modern methods are compared in terms of parameters, complexity, and reliability. Recent methods of water quality monitoring techniques are also reviewed to study any loopholes in modern methods. We found that CPS is suitable for monitoring water quality due to a good combination of physical and computational algorithms. Its embedded sensors, processors, and actuators can be designed to detect and interact with environments. We believe that conventional methods are costly and complex, whereas modern methods are also expensive but simpler with real-time detection. Traditional approaches are more time-consuming and expensive due to the high maintenance of laboratory facilities, involve chemical materials, and are inefficient for on-site monitoring applications. Apart from that, previous monitoring methods have issues in achieving a reliable measurement of water quality parameters in real time. There are still limitations in instruments for detecting pollutants and producing valuable information on water quality. Thus, the review is important in order to compare previous methods and to improve current water quality assessments in terms of reliability and cost-effectiveness.

## 1. Introduction

Water pollution is a detrimental issue that should be taken seriously by the government, private sectors, non-private sectors, and the public. It is because 70% of the earth is made up of water, and the human body is made up of more than 60% of water [1]. Apart from that, the main water supply in Malaysia is originated from 99% of surface water and 1% of groundwater [2]. World Health Organization (WHO) states that clean and safe water is important for drinking, household use, industries, and health, where polluted water and poor sanitation can cause transmission diseases such as cholera, diarrhea, hepatitis, skin infection, typhoid, and other health risks [3]. For instance, 2300 people were affected by drinking contaminated water, and as a result, an outbreak of a waterborne disease epidemic happened in Walkerton, Ontario, Canada, in 2000, which was sourced from cattle manure from a nearby farm [4,5]. Water pollution in Lake Toba in North Sumatra was also caused by household, industrial, agricultural, and public transportation wastes [6]. Thus, it has affected the tourism industry and local residents’ health [6].

Water is used for drinking, agriculture work, industry operations, and cleaning. Water needs to be clean and safe because contaminated water can endanger human and aquaculture ecosystems. Malaysia has abundant water resources, but the accelerating pace of industrial developments, urbanization, and population growth has impacted water quality. States with large numbers of industrial areas and factories, such as Selangor, Johor, Penang, and Perak, have high levels of river pollution compared to other states in Malaysia. Movement Control Order (MCO) was enforced from 18 March to 4 May 2020, when Malaysia was badly affected by COVID-19. Malaysia enforced ‘Ops Air Raya’, where two factories, a glove factory in Ipoh, Perak and an oil palm mill in Johor, had their operations suspended [7]. Thus, restricted business activities during MCO, which affect human mobility restrictions, and anthropogenic activities have directly shown a positive impact as they improved the quality index of water [8]. The three most common anthropogenic activities are industrial areas, sewage, agricultural activities, and animal husbandry activities. Kim Kim River in Johor also faces a problem of illicit untreated sewage wastewater discharge due to premise maintenance works [9]. In 2019, Kim Kim River had the worst river pollution due to illegal chemical waste dumping, and 6000 affected people were hospitalized. Apart from that, natural disasters such as floods can also pollute the water. In 2014, a huge flood occurred in Kelantan, Malaysia leading to murky and contaminated river. Due to the shortage of water supply during the flood, the victims had to use the polluted water for everyday usages, such as drinking, cooking, and bathing [10]. This situation caused the spreading of waterborne disease outbreaks such as diarrhea, malaria, and cholera. In other words, water pollution can cause deaths, unhealthy life, destruction of ecosystems, and contagious diseases.

**Figure 1:**
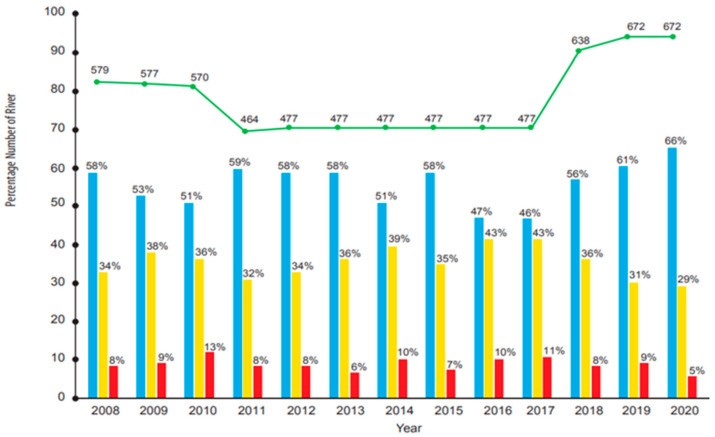
River Water Quality Trends from 2008 until 2020 year versus percentage number of rivers (%) in Malaysia [13]. Blue bar chart refers to unpolluted rivers; yellow bar chart refers to slightly polluted rivers; red bar chart refers to polluted rivers; green line shows total number of rivers.

Thus, a water quality monitoring system is considered the best solution to provide early assessments of contaminants in water. In Malaysia, monitoring water quality is normally performed by the government, assisted by private sectors, including weekly checks on groundwater, rivers, and lakes. Without adequate awareness of the importance of quality water management, Malaysia will face a water crisis by 2025 [2]. Groundwater is one of the critical sources of drinking water for households because 79% of the total population across 10 southeast Asia and pacific countries use groundwater which has common concerns related to pollution from unsafe sanitation, leading to dry season shortages and others [11]. Moreover, contaminants that lead to water pollution can be detected through physical, chemical, and biological properties, as shown in Table 1 [12]. These water quality properties are important to determine water suitability for human consumption and ecosystem health. River water quality status from 2008 until 2020 is depicted in Figure 1 [13]. A total of 672 rivers have been monitored regularly. In 2020, 443 (66%) rivers show good quality of water, 195 (29%) rivers are slightly polluted, and the remaining 34 (5%) rivers are polluted. Water samples were collected from designated stations for in situ and laboratory analysis to determine physical, chemical, and biological characteristics. The Water Quality Index (WQI) is used to determine the quality of water and to indicate the pollution level based on National Water Quality Standards for Malaysia (NWQS) (ANNEX). The six water quality parameters used to determine the water quality index are dissolved oxygen (DO), biological oxygen demand (BOD), chemical oxygen demand (COD), total suspended solids (TSS), ammonia, and pH value [14,15]. Figure 1 shows the water quality for rivers in Malaysia from 2008–2020. The quality of river water fluctuates across three types of rivers; unpolluted rivers, slightly polluted rivers, and polluted rivers. The highest percentage of polluted rivers is 13% (2010), and the lowest percentage of polluted rivers is 5% (2020).

Here, we review recent methods of monitoring water quality in terms of features and parameters used. Section 2 discusses various water quality monitoring methods where traditional methods are compared with modern methods. Various water quality monitoring methods in many countries are discussed in this section. Some methods have the potential to be repeated and enhanced to produce a better water quality monitoring system. The review also compares previous water quality monitoring systems from 2015 until 2022 to detect many types of contamination with different approaches. Water quality monitoring methods which are based on the Internet of Things (IoT), real-time monitoring, wireless sensor network (WSN), filtration including traditional methods, and optical techniques, are discussed in Section 3. The existing techniques of water quality monitoring systems show reliable data with efficient processes. Therefore, the main objective of this review is to study existing methods of monitoring water quality, such as real-time with IoT, virtual sensing, and cyber physical systems (CPS) based on time, instrumentation, types of water quality parameters, or contaminants. Then, the strengths and weaknesses of the methods can be identified. CPS can support real-time monitoring, and performance can be guaranteed in safety-critical applications [16]. We discuss essential components of CPS in water quality monitoring, such as its history, benefits, and working principles. Thus, we believe that CPS is a reliable technique for water quality monitoring systems.

## 2. Comparison of Various Water Quality Monitoring Methods

Water treatment plant and water distribution system have their specific water quality monitoring tools to detect contaminants and check the suitability of the water for drinking purposes. In order to develop robust and efficient techniques with minimum operating cost and energy, numerous sensing and monitoring analyses research have been conducted over the past decades. There are still some tool limitations for detecting pollutants. Thus, current water quality assessments need to be improved. Khatri et al. [17] proposed a water monitoring system using a Raspberry Pi-based hardware platform. The system used a python framework for the development of a graphical user interface (GUI) and fuzzy logic for decision making. Apart from that, another system [18] used wireless sensor networks to continuously monitor water quality in remote places. The wireless sensor network (WSN) system consists of three parts: data monitoring nodes, a base station of data, and a remote monitoring station. The software design used MATLAB to interact with the hardware at the remote monitoring station. Development of a water monitoring system used Arduino that interfaced with LabVIEW that controlled pH level, turbidity, and temperature. The data are displayed in a graphical user interface (GUI) [1]. Table 2 summarizes the existing water quality monitoring systems from 2015 until 2022.

Hu et al. [7] applied water quality monitoring sensors which were placed in a water distribution system (WDS). WDS is a system where water sources, tanks, and connections are represented by nodes and borders to indicate pipes between the nodes. The system could be well implemented in many applications such as the health monitoring industry, smart buildings, localization, estimation and prediction, and diagnosis of fault [7]. However, the system can create some problems and difficulties, such as data obtained can be very complex in nature, leading to uncertainty and high cost. Several types of water quality monitoring sensors were used to detect water quality parameters, and Arduino was also used to integrate with the sensors to display the data efficiently [8]. The method used Arduino to receive reading values from every sensor, and then the data were sent to the Raspberry Pi through the internet [8].

Another enhancement was made by Y. K. Taru et al. [1], where the system interfaced the Arduino with the LabVIEW, which increased the performance of data acquisition. The system was flexible and easy to operate and install. The application of fuzzy logic to make a decision making was also developed by Khatri et al. [17], where the fuzzy approach was developed in MATLAB, and a Python framework was used to calculate the water quality index. Apart from that, optical techniques based on light propagation theory are important to track down the location sources and timing of sewage contaminations in real-time field settings with minimal cost, easy-to-handle process, and high accuracy results. Statistical relations of optical properties of water samples, such as reflection, refraction, fluorescence, and absorbance spectra can be used to calibrate and discover sewage contamination by using optical spectroscopy. For instance, optical technique that has been used recently is a vibrational spectroscopy. The instruments used for vibrational spectroscopy are Infrared (IR) and Raman spectrometer [22]. IR and Raman Spectroscopy are two commonly used vibration spectroscopy techniques for chemical and biological analysis that allow rapid and simple non-destructive measurement of several parameters simultaneously [35]. The approach is widely utilized in the study of the liquid and gas phases of water as it is highly dependent on the sample’s physical state.

Furthermore, a fluorescence spectroscopy method is required for the rapid detection of three common pathogenic bacteria such as *E.coli*, *K. pneumonia*, and *S. aureus*, with high sensitivity and efficiency to maintain water quality [29]. LIF (laser-induced fluorescence) was conducted using a UV laser as an excitation light source to excite dilutions that contain bacteria, and a spectrometer was used to receive fluorescence emission spectra concurrently. This study also analyzed various bacteria concentration gradients and proved that a good linear relationship exists between the height of fluorescence peak and bacteria concentration. Inactive *E.coli* does not influence the fluorescence peak position compared to active *E.coli*. The peak height of fluorescence differs greatly because inactive bacteria cannot grow continuously. Five critical factors that need attention are water temperature, pH, DO, EC, and salinity levels. Then, a wireless multi-sensor system was proposed, where an ESP 32 Wi-Fi module and Wi-Fi access point (AP) were integrated and displayed in the ThingSpeak IoT platform to monitor water quality parameters of freshwater aquaculture [30]. The authors mentioned that in order to estimate the level of salinity, the EC level information was acquired from the EC sensor. High-sensitivity sensors were used to provide good data accuracy and reliability. Based on the viewpoint of smart sensor aquaculture, the technique provides a simple feature for set up and maintenance, more cost-effective, simultaneous on-site monitoring, and thus, the overall system is highly reliable to use.

### 2.1. Traditional Methods versus Modern Methods in Monitoring Water Quality

Traditional methods can be used to monitor water quality. It is based on sample collection on site, chemical, physical and microbiological analysis performed in a laboratory. This technique involves labor and is cost-intensive [17]. Outcomes from traditional methods normally can be accessed after a few days, whereas modern methods can produce output in real time. An example of the traditional methods is performed by Central Water Commission. Water samples are collected from specific locations within the processing and distribution system, and the samples are tested at well-equipped laboratories. Samples of raw water, filtered water, and treated water were analyzed, and water quality parameters such as pH, turbidity, and dissolved oxygen were estimated by using lab-based equipment [36]. Results can be questionable due to errors from field sampling and equipment miscalibration. Apart from that, the sampling method can be very time-consuming due to the complicated process. The disadvantages of the traditional method are that the system is not continuous and not reliable as human energy is used to handle work, and the testing frequency can be very low [36]. The analysis works are normally carried out by a skilled person with high accuracy parameter detection results. Apart from that, laboratory facilities and maintenance are expensive [32]. The traditional laboratory methods consume more time, are costly, use chemical materials, and cannot give real-time readings [22]. Hence, the analysis lacks the continuous monitoring of systems.

Amrita et al. [37] performed a survey on water quality analysis between traditional and modern methods. Modern methods have more benefits than traditional methods since modern methods can produce output results and analyze the water quality parameters in real time. After a quick identification of poor water quality, faster action can be taken to handle undesired substances in the water. The traditional methods can cause delay and manual errors, which possibly occurs during the processes [27]. The traditional methods are basically based on sampling and monitoring water samples [37], and the analysis is performed in a laboratory. Errors can occur while doing sample preparation in the laboratory. Amrita et al. [37] used the titration method to determine water quality parameters in traditional ways. The titration method is time-consuming as it cannot be carried out within a day. The titration method is used to determine the carbon dioxide level in a solution using sodium hydroxide. By using potentiometric, pH can be determined when there is an exchange of ions between the swollen layer and the H+ ions in the emf. The swollen layer was formed when the outer layer of the glass bulb was hydrated as the glass electrode was dipped in water [37]. The method was developed using wireless sensor nodes to monitor water quality. The system consisted of ten parameters which were monitored inside node boxes, and it was connected through Wi-Fi with the wireless sensor node. There was an access point to send data to the farmers. If any problem occurred, the alarming pattern was used [37]. In contrast to the modern method, where sensors can monitor water quality parameters more quickly and deliver better results, the manual method of assessing water quality in aquaculture cannot produce consistent results, and it requires more time and more manpower. Due to the time-consuming and complex setup, the contents of the water sample may change and, therefore, produce less valuable data for monitoring water quality [25]. Thus, implementing more sensors can enhance the functionality of water quality monitoring system that indirectly can help authorities to implement quick measures to improve water quality.

### 2.2. Methods of Monitoring Water Quality in Various Countries

Many water quality monitoring methods have been established in many countries recently. For example, a fully automated nitrate monitoring station was created using the optical sensor [38], where the applications of UV optical nitrate sensors on surfaces and groundwater were introduced. The results showed that most of the nitrate variation had been observed at or near the water edge, with annual maxima occurring in late winter/early spring between the months of August and November due to leaching from New Zealand agricultural land [38]. Apart from that, Li et al. [39] established a new multistage decision support system with a complex multi-criteria decision making (MCDM) for regional water quality assessment to overcome issues regarding regional water quality assessment. The system consisted of three stages. The first stage involved 21 multiple water quality indicators excluded the temperature indicators, and it used the probabilistic linguistic term set (PLTS) technique to process massive monitoring data. For the second and third stages, the proposed methods, such as regression-based decision-making trial and evaluation laboratory (DEMATEL), generated relative weight that considered the interrelationship of indicators and then further formed combined weight by balancing single-factor weight. For the final stage, a new LTS measure was demonstrated, and the fuzzy technique was extended to provide assessment findings. The proposed method was then used to investigate the water quality status of sixteen administrative districts in Shanghai, China [39].

Furthermore, Horvat et al. [40] developed an in-depth analysis of water quality in Lake Palic, Serbia. The analysis was performed by taking water quality measurements for 9 years, from 2011 to 2019. A principal component analysis (PCA) and machine learning classification methods were used to identify a seasonal feature of the water quality, and a fitted model was created via multivariate regression to determine water quality parameters [40]. Hasan et al. also used the multivariate analysis method to determine the quality of groundwater in the northeast part of Bangladesh [41]. Multivariate analysis was used in the system to interpret the water quality of selected pumps and to produce important results that could not be obtained from a cursory examination of the data.

N. Khatri et al. [42] determined the pollution levels in the River Sabarmati, Gujarat, India, and assessed the levels of multiple parameters with respect to drinking water standards. The system used water quality parameters such as pH, turbidity, total dissolved solids (TDS), total alkalinity, total hardness, chloride, ammoniacal nitrogen, biochemical oxygen demand (BOD), dissolved oxygen, and conductivity. The correlation analysis matrix showed that the basic ionic chemistry was influenced by these water quality parameters, especially pH, EC, TDS, K^+^, Na^+^, Mg^2+^, and SO_4_^2−^ [41]. In order to give an overall result of contamination in River Sabarmati, the Weighted Arithmetic Water Quality Index (WAWQI) and the Canadian Council of Ministers of the Environment Water Quality Index (CCMEWQI) were chosen. The results depicted differences between two indices in which the WAWQI showed the River Sabarmati was severely contaminated, not suitable to drink, and the condition of the river was even worse during the post-monsoon season, whereas the water quality ranged from ‘fair to marginal’ according to CCMEWQI [42]. Furthermore, the water quality of another river in India, River Netravati, was studied to determine heavy metal contaminations [43]. The technique used was similar to Horvat et al. and Hasan et al., where multivariate analysis was applied. Water and sediment samples were collected from ten locations along the Netravati River basin during the pre-monsoon season of 2019, and then hydrogeochemical features were investigated. Hydrogeochemical properties of water are important to determine the types of water used for domestic, industrial, and irrigation purposes. Metal contaminations were analyzed using multivariate techniques and environmental indices. Environmental indices are applied to indicate the status of water quality. In order to evaluate the water quality of the river, a comprehensive WQI method was adopted. Based on twelve measured water quality parameters, WQI was calculated for ten sampling stations. The analysis of total heavy metal concentrations and distributions proved that sediments were slightly contaminated with heavy metals which were due to increase in urbanization and agricultural practices, which changed the river hydrological regimes. The persistent exposure to pollutants, even at low concentrations, can cause changes in metabolic processes and changes in river community structure and thus, pose a serious threat of aquatic life [43].

### 2.3. Water Quality Monitoring Methods in Malaysia

Water quality has become a major concern in Malaysia. Aquaculture activities can impact the changes in water quality. Hettige et al. [44] performed research on water quality in the aquaculture sites at the Rawang sub-basin of the Selangor River. They quantified water quality parameters such as pH, dissolved oxygen (DO), ammoniacal nitrogen, turbidity, total suspended solids (TSS), chemical oxygen demand (COD), and biochemical oxygen demand (BOD) based on WQI. By using GIS (ArcGIS 10.2.1 software), the Inverse Distance Weighted (IDW) can be developed to determine the status of the water quality. The result indicates that the IDW can bolster identifying the potential aquaculture-impacted sites along the river.

A study on the quality of river water in Penang and Klang during (MCO) has been carried out by A. Najah et al. [45]. The impact of the MCO on the water quality index (WQI) in Putrajaya Lake was also examined by using four machine learning algorithms [45]. The water quality was improved during MCO as COD, BOD, and total suspended solid (TSS) were reduced. WQI Class I increased significantly from 24% in February 2020 to 94% in March 2020. Before MCO, the lake had only achieved WQI Class II for 94% in January and 76% in February 2020, respectively. During MCO in March 2020, the water quality of the lake experienced a rapid shift from WQI Class II to I. This condition is the best recorded WQI of Putrajaya Lake over the past 10 years. For WQI prediction, Multi-layer Perceptron (MLP) outperformed other models in predicting the changes in the index with a high level of accuracy. The sensitivity analysis results show that nitrogen content of ammonia (NH3-N) and COD plays a vital role and contribute significantly to predicting the class of WQI, followed by BOD, whereas the remaining three parameters; pH, DO, and TSS, do not contribute significantly towards WQI [45].

Abdul Maulud et al. [46] studied water quality in Kelantan River, Kelantan, Malaysia, during dry and rainy seasons by calculating the WQI based on the National Water Quality Standards for Malaysia (NWQS). The variables measured in the system are temperature, pH, TSS, DO, BOD, COD, ammonia nitrogen (AN), nitrate (NO_3_), phosphorus (P), and manganese (Mn). Othman et al. implemented a water quality monitoring system to maintain the aquaculture industry (Tilapia) in Malaysia and added the features of LABVIEW. The proposed system was in real-time, in which the data could be monitored continuously with the capability of recording and analyzing each reading in a more efficient way [47]. An alarm system was also available in the system to notify the users if any of the parameters deviated. The outcome of the developed system showed that the water quality for the tilapia industry could be measured by taking two parameters such as pH and temperature. The experiment shows that the percentage error between manual and automated measurements is less than 7% for the temperature parameter [47].

## 3. Common Methods of Water Quality Monitoring System

### 3.1. Virtual Sensing System

A virtual sensing system is basically enhanced from a fully physical system [32]. In contrast to a physical sensor, a virtual or soft sensor processes the accessible secondary data through models and enables the prediction of target parameters [32]. It converts several inputs from cheaper sensors and combines them to execute the outputs of more complex and expensive sensors. This model is constructed with three main approaches, which are knowledge-based, mechanism-based, and data-derived or machine learning methods. The soft sensing technique can be implemented as an alternative method to measure online water quality parameters, which are COD, BOD, chlorine, and total phosphorus [48]. Meanwhile, machine learning has the ability to extract informative data from an accessible database. Thus, it proves that this method is an ideal framework for virtual sensor applications. For example, the IBK algorithm machine learning (ML) based soft sensor model is an alternative method for estimating BOD level. It shows that the BOD soft sensors are efficient, reasonably accurate, and economical [49]. The system had been tested, validated, and verified with the sewage data from the water treatment plant and from the Ganges River. K-Nearest Neighbor (KNN) technique is another data-driven ML algorithm that proved to be an efficient method for COD prediction and evaluation in terms of response time and other performance matrices [48]. In the wastewater treatment plant, a few indicators, such as BOD and COD, are difficult to perform timely with hardware tools and hard to obtain accurate measurements [50]. The author proposed a soft measurement model construction by the lion-swarm-optimizer-based extreme learning machine (LSO-ELM). It can improve soft quality measurement in the wastewater treatment process because the method is able to achieve satisfied prediction accuracy [50]. Therefore, virtual sensing approach entirely promotes some benefits in cost and quality. However, the overall system is quite complex with the expensive sensor replacement possibility from cheaper sensors such as pH, temperature, DO, conductivity, and others.

**Figure 2:**
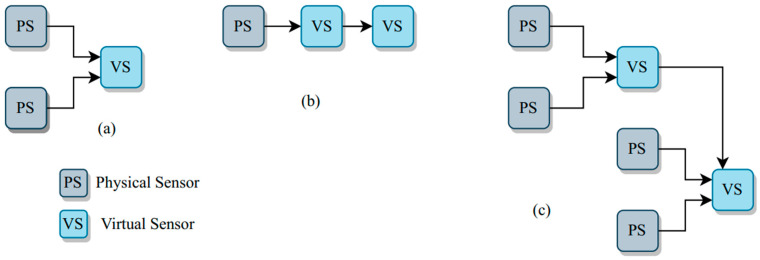
Three virtual sensor (VS) constellations: (**a**) VS based entirely on physical sensor (PS), (**b**) VS based only another VS and (**c**) VS based on both virtual and physical sensors [32]. Virtual sensor intercorrelates with data captured by physical sensors which are embedded into software applications to implement the algorithmic analytics from all the data sets given.

**Figure 3:**
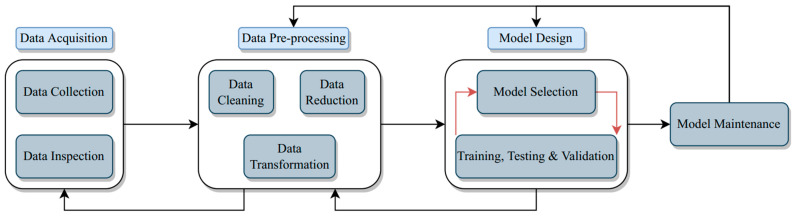
An overview of virtual sensing development steps [32].

Basically, there are three virtual-sensor (VS) constellations, including VS based entirely on a physical sensor, VS based only on another VS, and VS based on both virtual and physical sensors, as depicted in Figure 2. Virtual sensing intercorrelates with data captured by physical sensors, which are embedded into software applications to implement the algorithmic analytics from all the data sets given. VS is cheap as no equipment is needed to buy and maintain. It is ideal for high-frequency monitoring because it does not require a long chemical reaction process and can be easily scaled in many locations without extra investment. For virtual sensor development in water quality monitoring, there are four steps which are data acquisition, data pre-processing, model design, and model maintenance (Figure 3). Data collection is the first step to developing the data-derived virtual sensing and achieving the associated water quality targets [51]. Low-quality data lead to low-quality models. Data inspection is used to investigate the prominent data structure from data outliers, missing values, and others. The second step, data pre-processing, refers to data processing that includes typical data cleaning, transformation, and reduction. It can reduce the data size by redundant and non-relevant input reductions. Next, model design is very important in virtual sensing development as the model structure selection is task-dependent, and currently, there is no standard approach to perform this task. Normally, this step will start with a simple model type, performance verification, and model system improvement. The model complexity will gradually increase to obtain the expected outcome [52]. The last step is model maintenance. The model design that has been constructed and evaluated needs to be maintained and updated regularly as the data will change timely.

**Figure 4:**
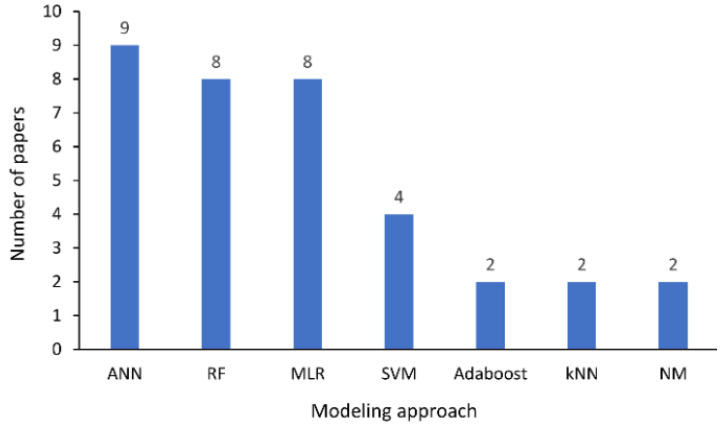
Machine learning (ML) Techniques used the most from 2019–2021 [32]. ANN refers to artificial neural network; RF refers to random forest; MLR refers to multiple linear regression; SVM refers to support vector machine; Adaboost refers to adaptive boosting; kNN refers to k-nearest neighbor and NM refers to numerical models.

Moreover, there have been several machine learning algorithms used for water quality monitoring systems in the past three years, from 2019 to 2021. According to the author, artificial neural network (ANN) modeling is the most-used ML modeling approach applied for water quality monitoring, as demonstrated in Figure 4 [53]. ANN techniques provide more accessible calibration and robustness capable of processing nonlinear and complex datasets and can provide satisfactory prediction results with a small data amount [53]. ANN modeling configurations can be used to predict BOD and identify the wastewater treatment plant performance processes [54]. The prediction models that are used to estimate BOD can save time and allow online control systems. Other ML techniques such as random forest (RF) and multiple linear regression (MLR) are normally used as the algorithm as it is simpler compared to other ML algorithms. The new generation of ML is hybrid models, which are developed from different conventional ML models and integrated with optimization methods. It is applied in order to achieve better performance and empower computation, functionality, and accuracy from the single model [55].

Data-driven virtual sensing technique needs to have several inputs and easily measurable parameters to construct prediction models. Input parameters such as pH, electrical conductivity (EC), temperature, turbidity, and DO can be used to measure output parameters such as total phosphorus (TP), sodium absorption ratio (SAR), total nitrogen (TN), magnesium absorption ratio (MAR) and residual sodium carbonate (RSC). For example, to predict the TP and TN, we need COD as one of the inputs. The sensing module placed in the water can convert the water quality parameters into an equivalent measurable electrical quantity that is transmitted to the coordinator module. Accurate and reliable sensors used are important as it affects the efficiency. In other words, the predictive accuracy of virtual sensing degraded gradually because of inappropriate input parameter sensor selection, insufficient sample numbers, process nonlinearity, and others.

### 3.2. IoT and Real-Time Implementation of Water Quality Monitoring

Internet of Things (IoT) can be applied in water quality monitoring systems in order to send data through the internet. For example, Pasika et al. [25] used IoT to transmit, gather and analyze information in real-time. The proposed system used multiple sensors such as pH sensors, turbidity sensors, water level, temperature, and humidity sensors and interfaced with the microcontroller unit, Arduino Mega. The system used the ThingSpeak application to send data to online storage, known as the ‘cloud’. The real-time algorithm to detect water quality is successfully developed in the proposed system, but it can be enhanced by adding more parameters to detect water quality, such as an oxygen reduction potential sensor or dissolved oxygen sensor [25].

Meanwhile, Mahajan et al. [27] used LEDs to reduce time delay for water quality detection. The system performed faster than existing systems. The system was able to inform the users to detect water quality immediately, but it did not analyze the parameters. To enhance the deficiency method by Mahajan et al. in 2020, Pujar et al. [56] also developed a water quality monitoring system using IoT where it applied statistical analysis in IoT. River Krishna, located in the Karnataka region, was chosen as a study area to develop the system. The system used multiple types of water quality sensors, and the statistical analysis was based on one-way and two-way analysis of variance (ANOVA). The result showed that one-way analysis was the most suitable analysis to be implemented with IoT [56].

In 2018, a real-time water quality monitoring system was developed using a low-cost wireless sensor network. In the system, the ammonia concentration in the water and pH and the temperature of the water were detected and monitored. When sensors were placed in the water, water quality parameters were detected and sent to the cloud through an ethernet shield via phone or computer. Data could be analyzed, and the alarm signal was sent to the users if any parameter values were out of their safety range [57]. Wireless Sensor Network (WSN) technology was used in [57] to provide real-time monitoring. The technology gave important information on water quality management to ensure the fertility of aquatic life and enhance human health. Meanwhile, Sabari et al. [58] designed a real-time water quality monitoring system with IoT. The system used several water quality parameters such as pH sensor, temperature sensor, turbidity sensor, and flow sensor. The system was interfaced with Arduino, and the data could be viewed through a Wi-Fi system [58]. The system was economical, convenient, and fast as it could automatically monitor the water at a low cost and with less human energy consumption.

In addition, various water quality monitoring systems (WQMS) with IoT integration have been reviewed by M. Monaj [33]. To build a smart freshwater pond for aquaculture with automatic maintenance and water quality monitoring, the authors proposed underwater sensors to continuously record parameter values in regular intervals with Arduino/Raspberry Pi module for processing and transferring data. Underwater sensors consisted of ammonia sensors which used AmmoLyt, nitrogen sensors, DO sensors, LM35 temperature sensors, and pH sensors. Traditional WMQS systems need to adjust the operation manually when there is a data mismatch, whereas the IoT-based WMQS system can easily maintain the correct values if any mismatch in the data is found [33]. Then, water quality prediction can be constructed using a prediction framework based on multi-source transfer learning (MSTL). The system effectively uses water quality information from multiple nearby monitoring points to enhance prediction accuracy [31]. The same types of sensors need to be used at different monitoring points in order to have the same input parameter. In contrast, traditional transfer learning prediction methods only use one monitoring point source of water quality information, which ignores any information near the monitoring points. They performed the experiment in Hong Kong to verify actual water quality by training several water prediction models using the adjacency effect to reduce prediction bias and improve prediction accuracy.

Furthermore, W. Hong [59] demonstrated water quality monitoring based on Arduino-based sensor systems. Temperature, pH, turbidity, and total dissolved solids (TDS) sensors were used and interfaced with Arduino. The results of this proposed system were taken for four weeks [59]. A simple prototype consisting of a microcontroller and multiple attached sensors was employed to conduct weekly on-site tests at multiple daily intervals. We found that the system worked reliably, but it relied on human assistance and was prone to data inaccuracies. However, the system provided a solid foundation for future expansion works of the same category to elevate the system to become Internet of Things (IoT) friendly. A recent study by Y. He [60] used embedded systems such as STM32F103VET6, serial communication module, and RS485 interface circuit to detect the aquaculture water parameter in real-time such as temperature, pH value, dissolved oxygen, turbidity, and other related information. Apart from that, Chang et al. [61] developed a system that used sensors to develop an unmanned surface vehicle (MF-USV) to avoid any obstacles and monitor the water quality system and water surface cleaning system. The obstacles can be any animals, plants, or things on the surface. The MF-USV consisted of several components that acted as autonomous obstacle detection to detect pH water and water surface cleaning. It could detect and collect the floating garbage on water and perform remote navigation control and real-time information display [61]. In the system, a pH sensor was used to detect the pH of the water before being analyzed in a laboratory. Although the system is in real time, the process consumes extra time for data analysis.

### 3.3. Cyber-Physical System

Cyber-Physical System (CPS) was firstly introduced by Helen Gill in 2006 at Natural Science Foundation, United States [62]. CPS is a system that incorporates physical components into a computational algorithm smoothly. CPS is the future of embedded systems. A full-fledged CPS is usually configured as a network of interacting components with physical input and output rather than as stand-alone devices, unlike embedded systems. After all, CPS offers more benefits as it uses a user-friendly decision support system such as fuzzy logic to overcome the complexity of data points that are generated from several sensor nodes or known as sensor arrays [63]. According to Lee [62], Wiener was the one who developed the CPS during World War II when he invented the technology of aiming and firing anti-aircraft guns. CPS is widely connected nowadays, such as in IoT, Industrial 4.0, Industrial Internet, and Machine-to-Machine. CPS can be used in many applications, such as healthcare applications where the system provides healthcare professionals and services to patients in real-time [64]. CPS also can be used in large commercial and residential buildings to provide efficient working and living conditions [64]. Z. Wang [16] has discussed the opportunities and challenges of CPS for water sustainability which include four factors; sensing and instrumentation; communications and networking; computing; and control. The CPS for water sustainability was further investigated by Imen et al. [65], where five level architecture in CPS was developed, such as smart connection level, data-to-information connection level, cyber level, cognition level, and configuration level towards smart and sustainable drinking water infrastructure management. Bhardwaj et al. [63] developed a water quality monitoring system that used CPS, which consisted of sensing and computing frameworks for computational modeling.

**Figure 5:**
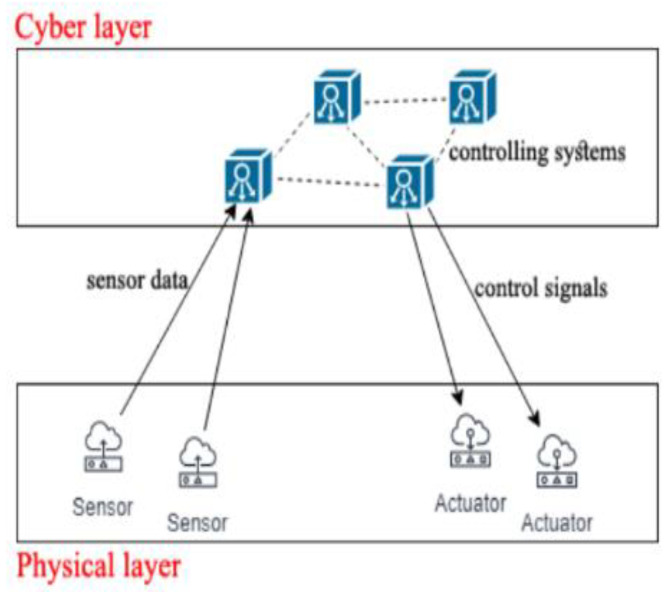
Overall Architecture of CPS [66]. Reproduced with permission from Mohamed, M.A.; Kardas, G.; Challenger, M; 2021.

CPS helps to monitor several parameters such as light, temperature, pH of water, and others. The first level of CPS architecture is a smart connection level, where, at this level, the selection of a correct sensor is important [65]. When the parameters of each sensor are read, the data are transferred to the controllers/software through wired/wireless communication [66]. In this state, a microcontroller such as Arduino is required to communicate with it. Then, a computational framework is needed to deal with the received data from sensors to make decision making, and finally, the data are sent to actuators through a communication system. The physical phenomena can change in return to make a feedback loop [66]. The overall system is shown in Figure 5.

**Figure 6:**
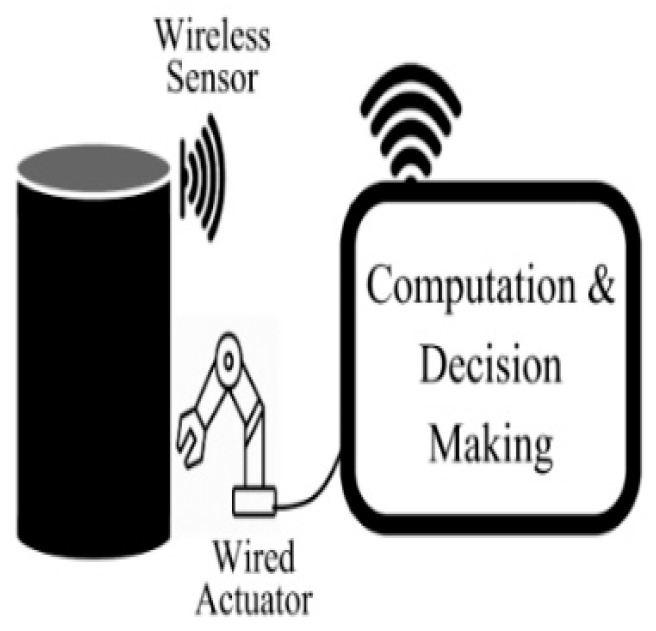
The heterogeneous components of CPS that connected through wired and wireless communication [64].

**Figure 7:**
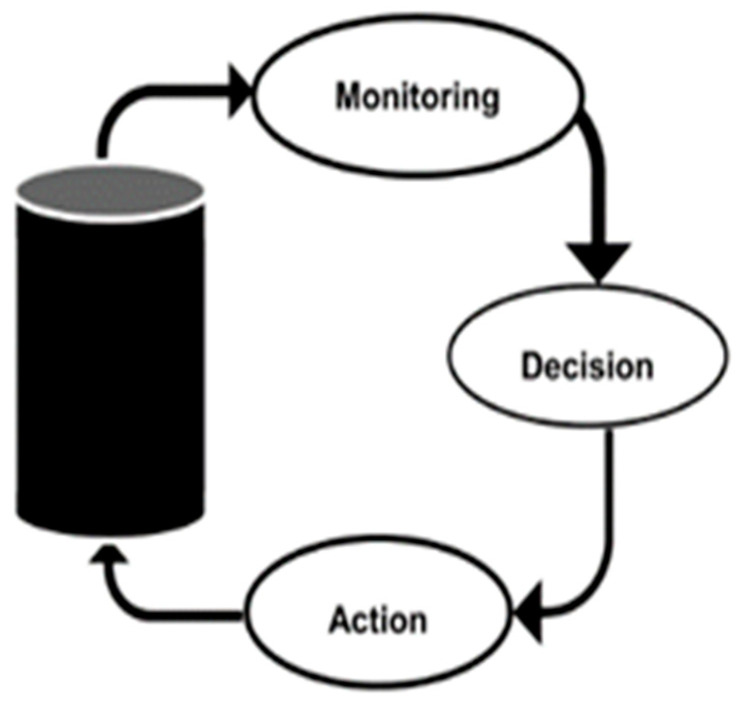
Control system of closed loop in CPS [64].

On the other hand, CPS consists of heterogeneous, distrusted components such as computing nodes, sensors, actuators, smart devices, and software [64]. In order to connect these components, wired and wireless connections are needed, as shown in Figure 6. In order to connect the cyber world with the physical world, sensors and actuators play a vital role in interfacing them, as sensors can monitor the physical world, whereas actuators manipulate the physical world [64]. It is basically based on configuration level, a feedback loop from a cyber system to a physical system [65]. Figure 7 depicts the operation of the feedback loop based on CPS. It consists of three main functions such as monitoring using sensors, making decisions using smart software, and applying actions using actuators [64].

Bhardwaj et al. [63] proposed a water quality monitoring system based on CPS. Their system consisted of three stages. The first stage involved designing the sensing framework, where five types of water quality sensors were chosen. Then, it used Arduino to control the sensors, and data from sensors were sent to a computer framework using C/C++ and Python. Fuzzy logic was applied in this system to make a reliable and efficient decision making of the system. Three membership functions (MFs), which consist of not acceptable (NA), adequate (ADE), and highly acceptable (HACC), were assigned with different ranges of water quality parameters from Fuzzy representation, shown in Table 3.

**Figure 8:**
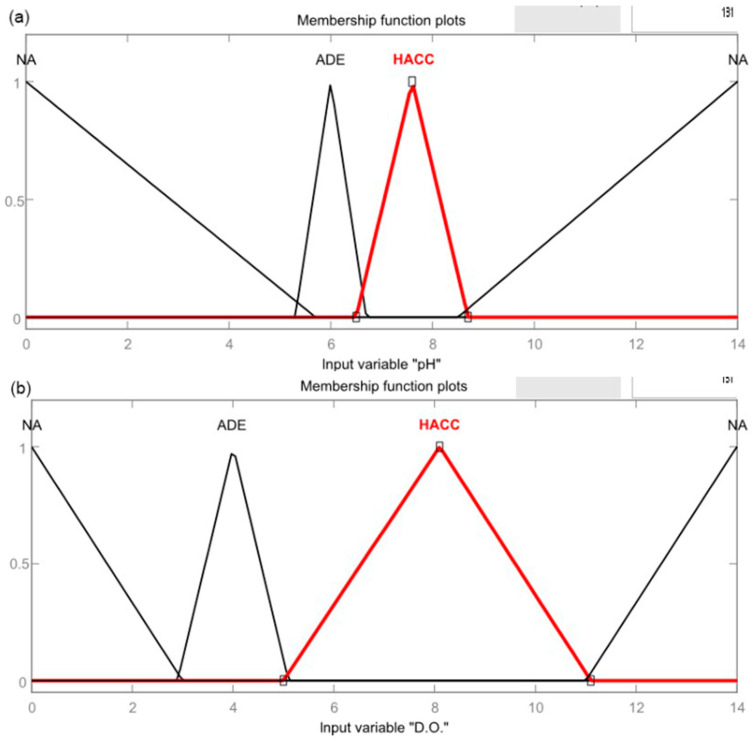
Membership function (MF) plots based on fuzzy rule for water quality parameters (**a**) pH and (**b**) DO [63].

Then, the water quality was decided based on rules: (1) if one parameter is NA, the water quality is NA, (2) If one parameter is ADE (provided that no parameter is NA), the water quality is ADE and (3) If all parameters are HACC, the water quality is HACC. Figure 8 shows the fuzzy rule applied in the system.

**Figure 9:**
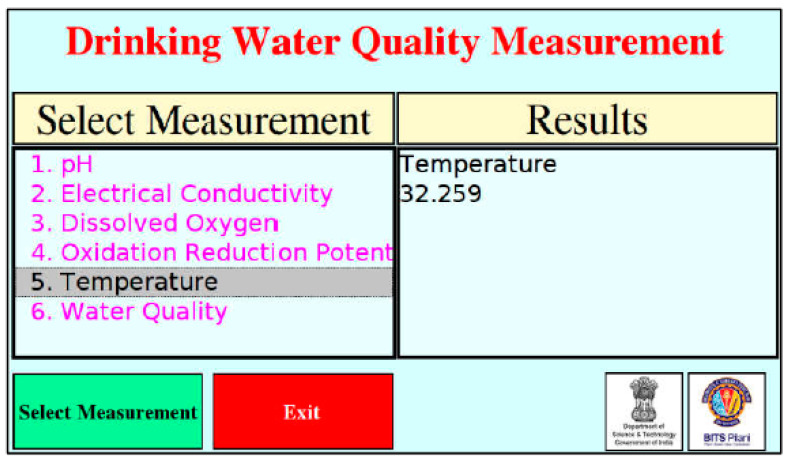
Graphical user interface (GUI) developed for water quality monitoring system [17].

A water quality monitoring system has also been developed using a similar concept from [13]. The system used Raspberry Pi as a microcontroller that interfaced directly with python. The system consisted of a graphical user interface (GUI) that was implemented on the Raspberry Pi board. The board served as an independent system where any computer was used. The system could observe more than three water quality parameters [17]. Figure 9 shows GUI, where the individual parameter to be measured can be selected, and the water quality can be checked.

Cyber-physical systems (CPS)s can be used to analyze water quality. CPSs are smart network systems that are operated with embedded sensors, processors, and actuators. It is considered an emerging technology and can be designed to sense and interact with the physical world, such as the water environment [16,63]. CPS systems have high autonomy, fast quality detection due to quick process of decision making, efficient and flexible. CPS also involves a stable, robust, scalable, and reliable process. The data analysis by CPS is precise and accurate. It is communicative where the CPS system can connect and share the data with entire water quality systems [67].

Engineers and scientists should clearly understand the concept of artificial intelligence, machine learning, neural networks, and other modern online technologies to apply CPS [68]. Mohamed et al. [66] proposed to design the CPS system that consisted of several complex software and hardware, with a high-level abstraction of the system. He suggested model-driven engineering, which was commonly used in the business domain for the development of software. CPS needs many developers from various background studies such as software engineering, electric and electronic engineering, computer science, and other sectors. It creates a communication challenge between developers as various tools and abstractions are implemented in each field.

### 3.4. Optical Techniques

Optical sensors and spectroscopic approaches are other examples of water quality monitoring techniques. Recently, smart sensing platforms can work together with electronics and optical sensors to improve and control the monitoring system. Electronics sensing is portable and simple to handle, whereas optical sensing does not affect water sample and provide higher accuracy result [69]. Optical sensors can monitor the total suspended solid (TSS) concentration from light transmission through water samples [69]. Light emitting diode (LED) acts as a transmitter to transmit light through suspended particles in water samples. Physical variables such as particle size, shape, suspended solid concentration (SSC), composition, and chemical properties affect light transmission through water samples. Examples of known optical sensors are the charge-coupled device (CCD) linear sensor, phototransistor, optical biosensor, fluorescence sensor, lasers, and others [70,71,72,73,74,75]. Apart from that, another optical fiber sensor was designed based on the principle of surface plasmon resonance (SPR) to monitor the interaction of biological molecules in real time without the need for labeling, separation, and purification [73]. The designed system is capable of measuring oil in wastewater at different concentrations with high accuracy, fast detection, good stability, easy operation, and allows online monitoring. Next, a low-cost autonomous optical sensor is devised to be environmentally robust, easily deployable, and simple to operate [74]. It consists of a multi-wavelength light source with two photodiode detectors that can measure the transmission and side scattering of the light in the detector head. Thus, the sensors can provide qualitative data on the changes in the optical opacity of the water. The optical colorimetric sensor (OCS) provides data on bulk water property changes, particularly opacity and color changes. This sensor also clearly provides valuable data related to turbidity events [74].

Meanwhile, spectroscopic techniques for detecting contaminants are continuously upgraded in terms of detection sensitivity, quantitatively and qualitatively. There are several methods of spectroscopy that have been analyzed for monitoring water quality, such as vibrational spectroscopy, light emission or luminescence spectroscopy, fluorescence spectroscopy, near-infrared (NIR) spectroscopy, and others [23,34,76,77,78,79,80]. The techniques are extremely sensitive by producing accurate detection results of matter composition and determining physical structures through light propagation. Transmission, absorption, and reflectance spectra of light in water allow determination of turbidity of the water, size of particles, and concentration of contaminants in the water. It is suitable for detecting contaminants because each type of molecule in water samples reflects, absorbs, or emits electromagnetic radiation from light sources and analyzes light intensity characteristics to quantify the composition of the sample. The spectrometer can be used to determine the particle composition and size distribution of samples from optical properties [79]. Spectroscopy normally uses a light source or a laser as an emitter and a detector or spectrometer for spectral analysis [81]. The approaches are simple, non-invasive, rapid detection, and pollution-free, as no chemical materials are involved [23].

Z. Shi [34] reviewed the applications of online UV-Visible spectrophotometers for drinking water quality monitoring and process control. Compared to conventional methods, online UV-Vis sensors can capture events and allow quicker responses to water quality changes. Water quality measurements such as color, dissolved oxygen carbon (DOC), total organic carbon (TOC), turbidity, and nitrate can be performed directly using built-in generic algorithms of the online UV-Vis instruments. Online UV-Vis spectrophotometers are effective and practical for continuously measuring water quality parameters, and they do not need physical filtration and low maintenance. Future works require early warning detections and real-time water process control systems for water quality management.

Next, the spectroscopic technique uses the interaction between scattered light and water samples to gain knowledge of the chemical and biological components in the water. NIR spectroscopy with 700 nm to 1200 nm wavelength is widely used for some physical and chemical characteristics identification [69]. Reflectance spectrum can be obtained from lake water and differs in the water quality based on the presence of algae. Furthermore, H. Zhang [80] studied online water quality monitoring simplification with UV-Vis spectrometry and an artificial neural network for river confluence near Sherfield-on-Loddon. Convolutional neural network (CNN) and partial least squares (PLS) methods are implemented to calculate water parameters and obtain accurate results. Two water quality parameter, total suspended solids (TSS) and total organic carbon (TOC), concentrations showed precise results using PLS and CNN models based on predicted experimental values and true values. TOC is used to monitor changes in organic contents as it measures the amount of carbon in pure water or an aqueous system, whereas TSS is a particle that is larger than 2 µm in the water. Particles smaller than 2 µm are known as total dissolved solids (TDS). Overall, the outcome of the study shows that the combination of spectroscopy with PLS and CNN models produces an accurate performance in estimating water parameters online. Apart from that, infrared (IR) spectroscopy uses a higher wavelength than UV-Vis with lower photon energy. The infrared spectra are classified into three categories which are near-infrared (NIR) (750–2500 nm), mid-infrared (MIR) (2.5–16 µm), and far-infrared (16–1000 µm) [82]. NIR spectrum is widely used for water quality analysis. NIR spectroscopy has been used for various experimental analyses, such as water monitoring for microalgae and extracellular polymeric substances in wastewater processes. Optical systems can provide valuable information on the composition and quality of water [82]. Various spectroscopic types with different features can determine chemical, biological, and physical components in the water by manipulating light characteristics, including transmission, absorption, reflectance, and fluorescence spectra.

## 4. Conclusions

In conclusion, water pollution is a detrimental issue that should be taken seriously by the government, non-public sectors, and society. In order to mitigate the issue, it is essential to have a reliable and continuous real-time water quality monitoring system that can provide useful output data and help authorities choose appropriate and fast actions. Thus, the review aims to investigate previous methods of water quality monitoring systems, compare traditional and modern methods and study different methods from various countries. Water quality monitoring methods such as the CPS approach, electronics sensing methods, virtual sensing system, IoT approach, and optical techniques are reviewed extensively. The review shows that CPS is relevant and acceptable to be used in water quality monitoring systems. Apart from that, CPS is a smart and reliable system where it can connect two worlds: (1) the physical world, such as sensors, environments, and humans, and (2) the cyber world, such as software and data. Indirectly, real-time monitoring of water quality can be achieved and offers the possibility of providing early warning in the water quality management system. Thus, water pollution can be detected, and the quality of water can be analyzed before it can be safely used by consumers. In the future, CPS technology can be combined with advanced optical techniques to produce high reliability and sensitivity because current existing monitoring methods have difficulties in obtaining an accurate measurement of water quality parameters in real-time and cost-effectiveness with continuous data measuring. Until now, there are some tool limitations to detect pollutants and require enhancement on existing water quality assessments.

## Figures and Tables

**Table 1:** Water Quality Properties.

Chemical Properties	Biological Properties	Physical Properties
Dissolved oxygen(DO), chemical oxygen demand (COD), biological oxygen demand(BOD)	Bacteria	Turbidity
pH level Ammonia Salinity Harness Organic Compounds Metals	Algae Viruses	Temperature Color Taste and Odor Suspended Solids Metals

**Table 2:** Previous works on water quality monitoring system.

No.	Water Quality Monitoring Method	Types of Contaminant/Water Quality Parameters	Real-Time Monitoring	References
1.	IoT environment such as Intel Galileo Gen 2 which acted as an interface to obtain data from multiple electronics sensors.	pH, dissolved oxygen concentration, turbidity, and temperature.	Yes	[19]
2.	Supervisory Control and Data Acquisition (SCADA) system was integrated with IoT technology to determine water contaminations, leakage in pipeline and automatic measure of several water parameters with Global System for Mobile Communication (GSM) module.	Temperature, color, turbidiy.	Yes	[20]
3.	Real-time bacteria sensor to detect four different types of specified water pollution locations. The result was compared with laboratory analysis. It demonstrates the benefit of the bacteria sensor over turbidity sensors in monitoring bacteria in drinking water as early warning of microbiological pollutions.	Bacteria or abiotic particles.	Yes	[21]
4.	Several technologies for water quality monitoring such as discontinuous sample-based methods for biological and non-biological contaminants. Then, for in-line sensor monitoring, there are sensor placement approach, microfluidic sensors, and spectroscopic techniques.	Biological: *Escherichia coli (E. coli),* Intestinal enterococci. Chemical: aldicarb, glyphosate, colchicines and nicotine. Physical: temperature, conductivity, pH, ORP and turbidity simultaneously.	No	[22]
5.	Near Infrared (NIR) reflectance spectroscopy to predict water quality in Aceh River based on salinity and total dissolved solids.	Total dissolved solids	No	[23]
6.	Toxicity tests to detect chemical contaminants specifically toxic and adenosine triphosphate (ATP) level method to indicate contaminants by microorganisms.	Toxicity level and microorganisms.	No	[24]
7.	IoT technology development such as ThingSpeak to monitor the water quality. Data from multiple sensors which are connected to Arduino is sent to cloud using ThingSpeak.	pH value, turbidity, level of water in the tank, temperature, and humidity.	Yes	[25]
8.	A rapid Ultraviolet (UV)/Visible (UV-Vis) spectroscopy method for water quality monitoring and the water is sourced from on-farm root vegetable washing processes. The measurement is based on UV-VIS absorbance and used statistical methods such as principal component analysis (PCA) and partial least squares (PLS) regression.	Physical: Suspended solids, pH, BOD, COD, color dilution. Chemical: Organic substances, nitrogen, phosphorus Biological: *E.coli*, coliform bacteria pathogenic microorganisms	No	[26]
9.	Biological and chemical contaminants in water where polymerase chain reaction (PCR) is a suitable method for bacteria detection in water samples, based on extraction and replication of deoxyribonucleic acid (DNA) fragment samples.	Microorganisms and viruses.	Yes	[24]
10.	IoT based system in water monitoring by adding LEDs. The LEDs lighted up depending on the range of water quality that was detected by several sensors. The system was connected to Raspberry Pi, programmed with Java.	pH, turbidity, chlorine, nitrate, and electrical conductivity.	Yes	[27]
11.	Surface-enhanced Raman Scattering (SERS) as new modern bacteria detection method, based on ultrasensitive vibrational spectroscopy in surface and water waters.	Bacteria	No	[28]
12.	An efficient bacterial rapid detection using laser-induced fluorescence (LIF) spectroscopy technology based on the fluorescence intensity ratio (FIR) and fluorescence intensity to retrieve the bacteria concentrations.	Bacteria: *E.coli*, *K.pneumonia*, *S.aureus*	Yes	[29]
13.	A wireless multi-sensor system by integrating the temperature, pH, DO, and EC sensors with an ESP32 Wi-Fi module platform to monitor water quality of freshwater aquaculture. The estimated salinity level is by EC level sensing data.	Temperature, pH, DO, electrical conductivity (EC), salinity level	Yes	[30]
14.	A multi-source transfer learning (MSTL) for water quality prediction and effectively used water quality information of multiple nearby monitoring points to improve the water prediction accuracy and reduce bias.	Water quality information such as DO, phosphate, water temperature, nitrite	Yes	[31]
15.	Virtual sensing system feasibility from physical sensor methods for water quality assessment and focused on the water use for agricultural purposes.	pH, turbidity, temperature, conductivity, DO, total phosphorus	Yes	[32]
16.	Various effective water quality monitoring system (WQSN) for fishponds using IoT and underwater sensors to record the parameter values continuously in the regular time interval using Arduino/Raspberry Pi board.	pH, DO, nitrogen, ammonia, temperature	Yes	[33]
17.	An online UV-Vis Spectrophotometer for drinking water quality monitoring and process control. The approach is reagent-free, does not require sample pre-treatments and can provide continuous and reliable water parameter measurement with quicker response compared to conventional techniques.	Color, dissolved organic carbon (DOC), total organic carbon (TOC), turbidity, nitrate	Yes	[34]

**Table 3:** The range of water quality parameters for each membership functions (MF): not acceptable (NA), adequate (ADE) and highly acceptable (HACC) [63]. Reprinted with permission from Bhardwaj, J.; Gupta, K.K.; Gupta, R.; 2018.

Parameters	NA	ADE	HACC	NA
pH	<5.7	5.3–6.7	6.5–8.7	>8.5
Dissolved oxygen	<3	2.9–5.1	5.1–11.1	>11
Electrical conductivity	<300	290–510	500–1050	>1000
Oxygen reduction potential	<550	530–670	650–820	>800
Temperature	<2	1.9–10	9–36	>35

## Data Availability

Not applicable.
